# Differences in Functional Performance and Minimal Detectable Change According to Levels of Ankle Plantar Flexor Spasticity in Patients with Chronic Stroke

**DOI:** 10.3390/jcm14207358

**Published:** 2025-10-17

**Authors:** SeungHeon An, DongGeon Lee, DongMin Park, Kyeongbong Lee

**Affiliations:** 1 Department of Gait Lab, National Rehabilitation Center, Seoul 01022, Republic of Korea; 2 Department of Physical Therapy, Sinsegae Rehabilitation Hospital, Changwon 51216, Republic of Korea; 3 Department of Physical Therapy, Zenith Rehabilitation Hospital, Seoul 04993, Republic of Korea; 4 Department of Physical Therapy, Kangwon National University, Samcheok 25949, Republic of Korea

**Keywords:** muscle spasticity, ankle joint, postural balance, gait, reproducibility of results, stroke

## Abstract

**Background/Objectives**: Ankle plantar flexor spasticity after stroke may limit mobility, especially during turning and multi-directional stepping. Evidence on performance differences and measurement properties across spasticity levels is limited. We examined whether performance on the Activities-specific Balance Confidence Scale (ABC Scale), Five Times Sit-to-Stand Test (5xSTS), Figure-of-8 Walk Test (F8WT), and Four-Square Step Test (FSST) differs by spasticity severity, and evaluated test–retest reliability, the intraclass correlation coefficient (ICC), the standard error of measurement (SEM), and the minimal detectable change (MDC). **Methods**: In an observational cross-sectional comparative study, 54 individuals more than 6 months post-stroke were classified into three groups by the Modified Ashworth Scale (MAS = 0, MAS = 1 − 1+, MAS ≥ 2). Participants completed the ABC Scale, 5xSTS, F8WT, and FSST. One-way analysis of variance with Bonferroni adjustment tested group differences. Reliability was quantified using ICC (2,1); SEM and MDC at the 95% confidence level indexed absolute reliability. **Results**: No significant differences were found for the ABC Scale or 5xSTS. F8WT and FSST differed by spasticity level (*p* < 0.05), with poorer performance in the highest-spasticity group versus no spasticity. ICCs were high across assessments. All SEMs were <20% of test–retest means, and all MDCs were <20% of maximum scores. **Conclusion**: Assessments that require directional change detected differences across spasticity levels, whereas balance confidence and repeated sit-to-stand did not. All measures showed acceptable relative and absolute reliability. Findings support selecting outcomes by spasticity severity and using SEM and MDC as reference values when interpreting change in stroke rehabilitation.

## 1. Introduction

Following a stroke, individuals with hemiparesis frequently experience impaired motor control on the paretic side, primarily due to muscle weakness and reduced coordination [1,2]. Rehabilitation strategies thus emphasize enhancing postural balance and functional mobility [3]. Post-stroke spasticity is commonly observed in both upper and lower extremities [4], limiting voluntary movement and functional performance [5]. In particular, spasticity in the ankle plantar flexors has been reported to compromise gait, contributing to reduced stride length, slower gait speed, and asymmetrical walking patterns [4]. These gait deviations often increase reliance on assistive devices or the non-paretic limb, leading to greater energy expenditure, fatigue, and fall risk [6]. These limitations may negatively influence well-being and overall life satisfaction. Thus, the reduction in muscle tone in the lower limb has been recognized as a fundamental therapeutic goal to improve ambulation in stroke rehabilitation [7,8].

Turning and directional changes are essential components of ambulation during daily activities, and more than two turns are required for every ten steps in typical home settings [9]. These demands tend to increase in restricted spaces or when navigating around obstacles, where motor impairments may further raise the risk of falls [10]. Ankle plantar flexor spasticity may interfere with straight-line walking as well as functional movements such as sit-to-stand transitions, directional changes, and obstacle clearance. Despite its clinical significance, differences in performance on these complex motor tasks according to plantar flexor spasticity remain insufficiently investigated.

Standardized assessments, including the Activities-specific Balance Confidence Scale (ABC Scale) [11], Five Times Sit-to-Stand Test (5xSTS) [12], Figure-of-8 Walk Test (F8WT) [13], and Four-Square Step Test (FSST) [14] are widely used to quantify balance, strength, and dynamic mobility in stroke rehabilitation. These tools have been shown to provide reliable assessments of motor function [15,16,17]. Additionally, they support the calculation of minimal detectable change (MDC), which serves as a critical threshold for identifying true functional change over time [18,19,20]. Importantly, MDC values should be interpreted using validated tools with established reliability to ensure clinical applicability [21,22].

Previous research has shown that assessment outcomes and corresponding MDC values in tools such as the Berg Balance Scale, Fugl-Meyer Assessment, Timed Up and Go Test (TUG), and 10-Meter Walk Test vary according to the degree of ankle plantar flexor spasticity [23]. These results suggest that reducing ankle spasticity may contribute to improvements in balance, motor control, and gait function [24,25]. Nevertheless, limited evidence is available regarding differences in performance on the ABC Scale, 5xSTS, F8WT, and FSST based on the severity of ankle plantar flexor spasticity in chronic stroke patients.

To assess complementary aspects of mobility and balance, we selected four assessments with established measurement properties. The ABC Scale indexes balance self-efficacy in daily activities and has acceptable reliability and validity in individuals with stroke [26,27]. The 5xSTS reflects lower-limb functional strength and the transfer component of mobility, with good reliability reported in chronic stroke [28]. The F8WT requires curved-path ambulation and turning, tasks that are frequently impaired after stroke and relevant to community mobility [29,30]. The FSST assesses multidirectional stepping and obstacle negotiation, demonstrating strong reliability/validity and clinical feasibility, including in patients with post-stroke [14,31].

Therefore, the present study aimed to determine whether performance on the ABC Scale, 5xSTS, F8WT, and FSST differs according to the level of ankle plantar flexor spasticity in individuals with chronic stroke. In addition, this study assessed the test–retest reliability (intraclass correlation coefficient, ICC) and MDC values of these tools. It was hypothesized that lower spasticity levels would be associated with higher balance confidence, better lower limb motor performance, and improved gait ability. Furthermore, it was expected that performance outcomes, ICC, and MDC values would differ according to the severity of ankle plantar flexor spasticity.

## 2. Materials and Methods

### 2.1. Participants

This study included individuals with chronic stroke who were diagnosed with hemiparesis and hospitalized in two rehabilitation centers, one located in Changwon and the other in Seoul. Eligible participants were those more than six months post-stroke and undergoing comprehensive inpatient rehabilitation, including medical management, nursing care, physical therapy, and occupational therapy. Recruitment was conducted over a four-week period through bulletin board postings within each facility.

All participants received a detailed explanation of the study objectives and procedures. Only those who voluntarily agreed to participate and provided written informed consent were included. The inclusion criteria were: (1) diagnosis of stroke confirmed by a physician at least 6 months earlier; (2) ability to walk independently for at least 10 m [32], (3) Functional Ambulation Category score of 3 or higher [33], (4) score of 24 or higher on the Korean version of the Mini-Mental State Examination; (5) composite spasticity score of 10 or greater for the ankle plantar flexors [34], and (6) age between 20 and 65 years. Exclusion criteria included the following: (1) current use of medications affecting balance; (2) presence of orthopedic conditions involving the lower limbs; (3) unstable cardiopulmonary status; (4) or cognitive impairment severe enough to interfere with comprehension of the study protocol.

The required sample size was determined using G*Power version 3.1.9.7. Based on a previous study employing the same dependent variables [35], a one-way ANOVA model with three groups, significance level (α) of 0.05, statistical power (1–β) of 0.80, and a large effect size (f) of 0.59 was assumed [36], yielding a minimum target of 33 participants. To account for potential dropouts, 60 individuals were initially recruited. After excluding participants who withdrew from the study (*n* = 2), missing data (*n* = 1), emergency discharge (*n* = 1), and other reasons (*n* = 2), a final sample of 54 participants was included in the analysis.

### 2.2. Procedure

This study was designed as an observational, cross-sectional comparative investigation to examine differences in balance confidence, lower limb strength, and gait ability according to the severity of ankle plantar flexor spasticity in individuals with chronic stroke. All data on participant demographics and functional outcomes were collected by three licensed physical therapists, each with over 20 years of clinical experience in neuromuscular rehabilitation.

Before the assessment, ankle plantar flexor spasticity on the paretic side was assessed using the Modified Ashworth Scale (MAS), and participants were categorized into three groups based on MAS scores: MAS = 0, MAS = 1 ~ 1+, MAS ≥ 2 [23]. To evaluate the test–retest reliability of the four assessment tools, measures were taken to minimize potential learning effects for both assessors and participants. Each evaluator assessed at least two different participants per session, and retesting was conducted seven days after the initial evaluation to ensure consistent results across the two measurement sessions [19].

All assessments were conducted under the supervision of an additional staff member who remained in close proximity to ensure participant safety without interfering with performance. To minimize fatigue, participants were given 2–3 min of rest between each test. The order of the assessments was randomized for each participant, and all tests were completed within one or two consecutive days.

### 2.3. Measurements

The spasticity of the ankle plantar flexor muscles on the hemiparetic side was measured using the MAS. Inter- and intra-rater reliability of this scale has been reported as moderate to good, with weighted kappa coefficients between 0.45 and 0.64 [37].

Balance confidence and fear of falling were measured using the ABC Scale, which consists of 16 items, each rated from 0 (no confidence) to 100 (complete confidence). The total score is calculated as the mean of the 16 item scores and is expressed as a percentage (0–100%), with higher scores indicating greater balance confidence [11]. A concise summary of the scoring system is presented in Table 1. Higher scores indicate greater balance confidence. In individuals with chronic stroke, test–retest reliability has been reported as ICC = 0.85 [26].

Lower limb strength, transitional movement ability, and functional balance were evaluated using the 5xSTS. Participants were instructed to rise from a chair with a backrest and no armrests five times consecutively, as quickly as possible, while keeping their arms crossed over the chest and refraining from using their upper limbs. Seat height was standardized for each participant by measuring lower-leg (popliteal) height and adjusting the chair so that the starting position approximated 90° of knee flexion with feet flat on the floor; the same seat height was used for the 7-day retest. The time required to complete the task was recorded in seconds. The inter-rater reliability of the 5xSTS has been reported as ICC = 0.99 [38].

Gait ability was assessed using the F8WT and the FSST. For the F8WT, participants walked around two markers placed 1.52 m apart in a figure-eight pattern, with an additional 0.61 m of walking space on either side. Participants began at the midpoint, circled the left marker counterclockwise, returned to center, then circled the right marker clockwise. Time was recorded from the initial step to a complete stop at the starting point [13]. The F8WT has shown excellent inter-rater reliability, with an ICC of 0.99 [39]. The FSST involved stepping over four cylindrical rods (90 cm in length and 1 cm in diameter) arranged in a cross configuration on the floor. Participants moved in a predefined sequence: forward, right, backward, left, then reversed the sequence to return to the starting point. Time was recorded from the first step until the participant completed the final step back into the starting square [14]. If a participant touched the rods, stepped outside the boundary, failed to place both feet within a square, or required assistance, the trial was repeated. The participants were instructed to perform the task as quickly as possible while maintaining a forward-facing direction. The inter-rater reliability of the FSST has also been reported as ICC = 0.99 [40].

These tests primarily assess lower-limb function and dynamic balance without direct upper-limb support. The 5xSTS is performed with the arms crossed over the chest, and the F8WT and the FSST are performed without upper-limb assistance. Representative values from community-dwelling healthy adults are presented for reference only; no statistical comparisons with the study sample were performed.

### 2.4. Statistical Analysis

All statistical analyses were conducted using SPSS for Windows, version 29.0 (IBM Corp., Armonk, NY, USA). The Shapiro–Wilk test was used to verify the normality of the data. Descriptive statistics and frequency analysis were applied to summarize participant characteristics. To examine differences in performance on the ABC Scale, 5xSTS, F8WT, and FSST according to the severity of ankle plantar flexor spasticity, one-way analysis of variance was performed, followed by Bonferroni-corrected post hoc comparisons.

Test–retest reliability was assessed using ICC (2,1). An ICC of 0.75 ~ 0.90 was interpreted as good reliability, and >0.90 as excellent reliability [41]. The standard error of measurement (SEM) and the minimal detectable change at 95% confidence (MDC95) were calculated as SEM = SD × √(1 − ICC) and MDC95 = 1.96 × SEM × √2 [21,22]. An SEM <20% of the test–retest mean was considered acceptable. For interpretability, MDC95 was judged acceptable when <20% of the instrument’s maximum score for bounded scales (ABC = 100); for time-based tests without a fixed upper bound (5xSTS, F8WT, FSST), MDC95 was reported in seconds and as MDC% relative to the group mean, with MDC% ≤20% interpreted as low measurement error [18,20,42]. A significance level of α = 0.05 was set for all statistical tests.

## 3. Results

A total of 54 participants were included in the analysis, with a mean age of 60.3 years and a mean post-stroke duration of 26.2 months. Detailed characteristics, including stroke type, affected side, cognitive function, use of walking aids, and general performance outcomes, are presented in Table 2.

**Figure 1:**
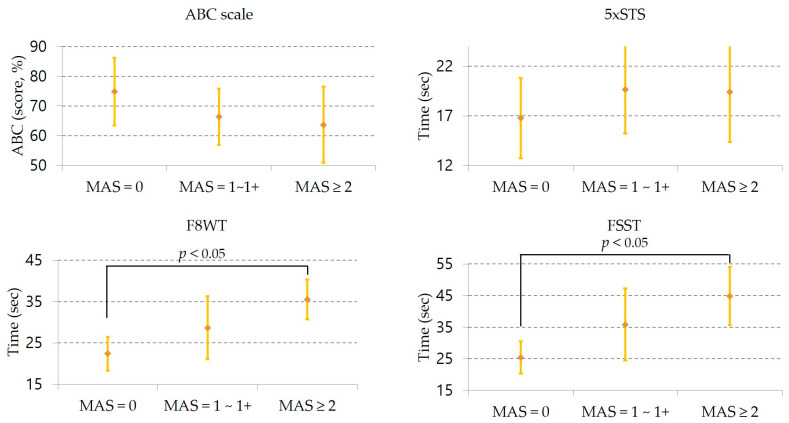
Functional performance across ankle plantar flexor spasticity levels. Group means (95% confidence intervals) for the ABC Scale, 5xSTS, F8WT, and FSST across ankle plantar flexor spasticity levels Significance are presented only for F8WT and FSST, indicating Bonferroni-adjusted pairwise differences. For time-based tests, lower values indicate better performance; the ABC scale is expressed as a score, percentage (0–100). Abbreviations: MAS, modified Ashworth scale; ABC scale, activities-specific balance confidence scale; 5xSTS, 5-times sit to stand test; F8WT, figure of 8 walk test; FSST, four square step test.

No significant differences were observed among the three groups in ABC Scale and 5xSTS. However, statistically significant group differences emerged in the F8WT (*p* = 0.041) and FSST (*p* = 0.049), with post hoc analyses indicating poorer performance in the MAS ≥ 2 group compared to the MAS = 0 group (Table 3). This pattern indicates an association between higher spasticity levels and slower performance on tasks requiring turning and multidirectional stepping (F8WT, FSST), whereas no clear association was observed for the vertical transfer task (5xSTS). For the ABC and 5xSTS, group means followed the expected direction across spasticity levels (higher ABC and shorter 5xSTS times with lower spasticity), but these differences did not reach statistical significance. Between-group differences are illustrated in Figure 1, which presents group means with 95% confidence intervals for all outcomes.

In Table 4, the ABC Scale, 5xSTS, F8WT, and FSST demonstrated good to excellent test–retest reliability across all levels of ankle plantar flexor spasticity. ICC values ranged from 0.88 to 0.98, reflecting strong measurement consistency regardless of spasticity severity.

The SEM values for all assessments were less than 20% of their respective mean scores across all groups, and MDC values remained below 20% of the maximum obtainable scores (Table 5). These results support the reliability and clinical applicability of the ABC Scale, 5xSTS, F8WT, and FSST for detecting meaningful functional changes across varying levels of spasticity.

## 4. Discussion

This study aimed to quantitatively investigate differences in balance confidence, lower limb strength, and gait ability based on the severity of ankle plantar flexor spasticity in individuals with chronic stroke. Individuals with more pronounced spasticity exhibited significantly diminished performance in tasks involving complex gait components, such as directional changes and obstacle clearance. Conversely, outcomes related to balance confidence and repeated sit-to-stand transitions did not differ significantly across spasticity levels, suggesting that these domains may be less sensitive to localized ankle impairments. These findings emphasize the selective impact of plantar flexor spasticity on dynamic gait ability and reinforce the importance of incorporating spasticity-targeted strategies within gait rehabilitation programs for individuals with chronic stroke. Among the four assessments, the F8WT and the FSST showed significant between-group differences between spasticity-level groups, whereas the ABC Scale and the 5xSTS did not. This pattern indicates better discriminative performance for measures that require turning and multidirectional stepping in ambulant chronic stroke patients.

In practical terms, gait rehabilitation can prioritize task-oriented practice that reflects the demands taken by our measures. Circuit class training that incorporates stepping and turning has been shown to improve gait-related mobility after stroke [43]. Curved-path/turning practice is supported by training studies using the F8WT and reflects community mobility demands [44]. Obstacle negotiation and gait adaptability trainings can be delivered overground or via augmented-feedback/treadmill platforms that have feasibility and gains in walking adaptability or obstacle avoidance [45,46]. Treadmill training with or without body-weight support can yield small, short-term improvements in walking speed or endurance [47].

Balance confidence, as assessed by the ABC Scale, did not differ significantly according to the severity of ankle plantar flexor spasticity. This result is in agreement with previous research reporting no group differences in ABC scores, postural sway, ankle proprioception, or passive dorsiflexion range of motion among individuals with stroke exhibiting varying degrees of ankle spasticity [24]. One possible explanation is that postural stability in chronic stroke patients may depend more on visual input than on local spasticity [48]. Additionally, compensatory strategies involving the hip and knee joints, as well as increased reliance on the non-paretic limb, may contribute more substantially to maintaining balance [49,50]. These results suggest that self-perceived balance confidence may be influenced by a combination of psychological factors, such as fear of falling, and broader motor control mechanisms, rather than being directly determined by distal spasticity alone.

Although ankle plantar flexor spasticity may reduce joint mobility and compromise plantar stability during transitional movements, no significant differences were found in 5xSTS performance across spasticity levels. This outcome suggests that the test is more strongly influenced by proximal muscle strength and trunk control than by ankle function. Given its emphasis on rapid, repeated vertical transitions, the 5xSTS primarily engages hip and knee musculature, with limited demand on the ankle joint. Previous studies have reported similar findings, showing no significant associations between 5xSTS completion time and ankle dorsiflexor or plantar flexor strength on the paretic side [38,51], while performance was more closely linked to the strength of the paretic hip and knee muscles [52]. These findings imply that, although the 5xSTS is a reliable tool for evaluating lower limb strength, it may lack sensitivity in detecting the functional impact of ankle spasticity. Stroke patients often compensate for distal impairments by recruiting proximal muscle groups, which may account for the relatively stable performance across varying spasticity levels. Accordingly, incorporating complementary assessments that challenge distal control may enhance the clinical evaluation of motor deficits and support more targeted intervention planning.

Unlike other assessments, the F8WT assesses both linear gait and directional changes, providing a more comprehensive evaluation of dynamic gait ability. The significant differences observed across groups in this study suggest that ankle plantar flexor spasticity may substantially limit both gait velocity and turning efficiency. Spasticity in the paretic ankle has been identified as a major constraint on functional ambulation in individuals with stroke [4,53]. Prior studies have demonstrated strong correlations between the torque output of the medial gastrocnemius and the time required to complete mobility tasks such as the TUG, indicating the importance of plantar flexor strength and motor control in facilitating efficient movement [54,55]. These muscles are responsible for generating the majority of the propulsive force during the push-off phase, thereby governing forward limb progression and overall gait speed [56]. However, premature or exaggerated activation due to spasticity may disrupt normal timing and coordination, resulting in decreased symmetry and locomotor efficiency. Furthermore, plantar flexor spasticity has been shown to account for a meaningful proportion of gait performance variance in stroke [57], reinforcing its clinical significance. Collectively, these findings highlight the value of addressing ankle spasticity as a therapeutic target in gait rehabilitation and support its inclusion as a key factor when selecting outcome measures and evaluating treatment responses.

The FSST, which evaluates dynamic balance and multi-directional stepping ability, showed marked variability in performance depending on the level of ankle plantar flexor spasticity. In particular, participants with severe spasticity exhibited the greatest measurement error, indicating that spasticity may substantially interfere with the rapid and coordinated limb movements required by the task. This is consistent with a previous report emphasizing the sensitivity of FSST in identifying dynamic balance impairments among individuals with post-stroke lower-limb dysfunction [31]. The complexity of the test, involving continuous directional changes, may exacerbate the neuromuscular challenges posed by distal spasticity. Clinically, these findings highlight the FSST as a valuable tool for detecting functional limitations in patients with elevated spasticity levels. Nevertheless, to guide more individualized intervention planning, clinicians are encouraged to supplement this assessment with additional measures that specifically target distal motor control.

All clinical assessments in the present study demonstrated high test–retest reliability across levels of ankle plantar flexor spasticity, supporting their clinical applicability for evaluating gait and balance in individuals with chronic stroke. These findings are consistent with prior studies reporting strong reliability for each tool in patients with stroke [26,38,39]. While ICCs indicated high relative reliability, absolute reliability was further evaluated using SEM and MDC, which help quantify measurement error and the minimum change needed to exceed it with 95% confidence [20,21,22].

In this study, all SEM values were below 20% of the test–retest mean scores, indicating acceptable measurement precision. The highest SEM on the ABC Scale was found in the no-spasticity group, suggesting variability in balance confidence even without notable motor impairment. For the 5xSTS, SEM values, supporting the notion that this test reflects proximal strength rather than distal motor involvement. In contrast, the highest SEMs for the F8WT and FSST were observed in the moderate and severe spasticity groups, likely due to the increased motor demands of complex gait tasks.

All MDC values also remained under 20% of each tool’s maximum possible score, further confirming their suitability for detecting clinically meaningful change. The FSST and F8WT exhibited the largest MDCs in patients with greater spasticity, indicating these assessments are particularly responsive to impairments in dynamic gait control. These findings emphasize the importance of selecting appropriate outcome measures based on spasticity severity and support the use of MDC as a reference value for evaluating therapeutic change and guiding rehabilitation planning.

This study has several limitations. First, spasticity was assessed using the MAS, a measure known to be influenced by examiner subjectivity, which may limit the precision of spasticity classification. Second, the study included only ambulatory patients with chronic stroke, and performance was evaluated primarily by task completion time, not by qualitative aspects of movement execution. Third, test–retest reliability and responsiveness were examined over a short-term interval, without evaluating long-term functional changes or intervention effects. Future studies should adopt longitudinal designs to assess whether changes in MDC values are associated with clinical improvement following treatment. Despite these limitations, the present findings suggest that MDC can be a valuable index for detecting meaningful functional change under specific clinical conditions. Additionally, ankle plantar flexor spasticity appears to play a key role in interpreting assessments of lower limb strength and gait ability in stroke rehabilitation, and the rehabilitation examples were provided for context; we did not test these interventions or quantify responsiveness in this cross-sectional study.

## 5. Conclusions

Chronic stroke patients with higher levels of spasticity demonstrated lower performance in tasks involving complex gait and dynamic balance, whereas no significant differences were observed in measures of balance confidence and lower limb strength. All measures demonstrated high test–retest reliability, supporting their use in clinical practice. Clinically, measures requiring turning and multidirectional stepping better discriminated performance between spasticity-level groups, whereas balance confidence and lower limb strength did not in ambulatory individuals with chronic stroke. For repeated assessments, observed changes should be evaluated against the SEM and MDC for each measure; these cross-sectional findings reflect discrimination, not responsiveness. These findings support selecting impairment-specific outcome measures and using measurement-error indices to interpret change. Further studies are needed to determine responsiveness and treatment effects following spasticity-targeted rehabilitation.

## Figures and Tables

**Table 1:** The Activities-specific Balance Confidence Scale scoring and reporting.

Component	Description	Reporting Unit	Interpretation
Items	16 daily balance activities	0–100 each	0 = no confidence; 100 = complete confidence
Total score	Mean of 16 item scores	Percentage (0–100%)	Higher = greater confidence

Note. Scores on the Activities-specific Balance Confidence (ABC) Scale are expressed as percentages (0–100%); higher scores indicate greater balance confidence.

**Table 2:** Clinical characteristics and outcome measures of the subject (*n* = 54).

Parameters	Values
Age (year)	60.33 ± 14.48
Sex (male/female)	32 (59.3)/22 (40.7)
Duration (months)	26.19 ± 9.09
Diagnosis (infarction/hemorrhage)	34 (63)/20 (37)
Paretic side (left/right)	25 (46.3)/29 (53.7)
MMSE-K (score)	25.87 ± 1.66
Use of walking aid	
None/Single point cane/Quadri-pod cane	15 (26.8)/29 (51.8)/11 (19.6)
FMA-L/E (score)	25.87 ± 6.04
BBS (score)	44.49 ± 12.29
10mWT (m/s)	0.63 ± 0.26

Note: Continuous variables are presented as mean ± standard deviation. Categorical variables are expressed as number (percentage). Abbreviations: MMSE, mini mental status evaluation-Korean version; FMA-LE, Fugl-Meyer assessment-lower extremity; BBS, Berg balance scale; 10mWT, 10 m walk test.

**Table 3:** Comparison of functional performance evaluation based on ankle plantar flexor tone (*n* = 54).

Measures	Subgroups with Ankle Plantar Flexor Tone Mean ± SD (Min ~ Max)			F	Df	*p*	Post Hoc
	MAS = 0 (*n* = 16) (A)	MAS = 1~1+ (*n* = 23) (B)	MAS ≥ 2 (*n* = 15) (C)				
ABC scale (score, %)	74.81 ± 23.26 (31~97)	66.41 ± 23.09 (23~99)	63.67 ± 25.34 (10~88)	0.951	2	0.393	ns
5xSTS (sec)	16.77 ± 8.24 (8.03~43.80)	19.67 ± 10.91 (9.93~46.72)	19.41 ± 9.98 (7.54~44.61)	0.451	2	0.639	ns
F8WT (sec)	22.40 ± 8.30 (13.06~38.77)	28.67 ± 18.62 (15.03~91.31)	35.50 ± 9.57 (23.92~61.71)	3.409	2	0.041	A ∣ C
FSST (sec)	25.42 ± 10.42 (13.87~49.77)	35.85 ± 27.96 (14.76~140.11)	44.87 ± 18.09 (10.38~85.73)	3.205	2	0.049	A ∣ C

Note: Values are presented as mean ± standard deviation. *p* < 0.05. Abbreviations: MAS, modified Ashworth scale; ABC scale, activities-specific balance confidence scale; 5xSTS, 5-times sit to stand test; F8WT, figure of 8 walk test; FSST, four square step test; ns, not significant.

**Table 4:** Test–retest reliability of functional performance assessments based on ankle plantar flexor spasticity (*n* = 54).

Measures	Subgroups with Ankle Plantar Flexor Tone								
	MAS = 0 (*n* = 16)			MAS = 1~1+ (*n* = 23)			MAS ≥ 2 (*n* = 15)		
	1st	2nd	ICC	1st	2nd	ICC	1st	2nd	ICC
ABC scale (score, %)	75.00 ± 24.18	74.63 ± 23.55	0.90 (0.75~0.96)	66.09 ± 24.94	66.74 ± 21.91	0.95 (0.90~0.98)	64.27 ± 27.28	63.07 ± 23.71	0.96 (0.90~0.98)
5xSTS (sec)	16.84 ± 8.66	16.69 ± 7.95	0.96 (0.90~0.98)	19.64 ± 11.12	19.70 ± 10.78	0.98 (0.96~0.99)	20.34 ± 10.54	18.48 ± 9.43	0.96 (0.77~0.98)
F8WT (sec)	22.95 ± 8.58	21.85 ± 8.12	0.96 (0.89~0.99)	29.21 ± 18.42	28.14 ± 19.20	0.95 (0.90~0.98)	35.74 ± 8.92	35.27 ± 10.74	0.88 (0.69~0.96)
FSST (sec)	25.44 ± 10.88	25.39 ± 10.26	0.94 (0.84~0.98)	36.60 ± 27.70	35.09 ± 28.43	0.98 (0.95~0.99)	46.21 ± 18.50	43.53 ± 18.68	0.89 (0.71~0.96)

Note: Values are presented as mean ± standard deviation. Abbreviations: MAS, modified Ashworth scale; ICC, intraclass correlation coefficient; ABC scale, activities-specific balance confidence scale; 5xSTS, 5-times sit to stand test; F8WT, figure of 8 walk test; FSST, four square step test.

**Table 5:** SEM and MDC of functional performance assessments based on ankle plantar flexor spasticity (*n* = 54).

Measures	Subgroups With Ankle Plantar Flexor Tone					
	MAS = 0 (*n* = 16)	MAS = 1~1+ (*n* = 23)	MAS ≥ 2 (*n* = 15)	MAS = 0 (*n* = 16)	MAS = 1~1+ (*n* = 23)	MAS ≥ 2 (*n* = 15)
	SEM			MDC		
ABC scale (score, %)	7.35	5.16	5.06	20.37	14.30	14.02
5xSTS (sec)	1.64	1.54	1.99	4.56	4.26	5.51
F8WT (sec)	1.66	4.16	3.31	4.60	11.52	9.17
FSST (sec)	2.66	3.95	5.99	7.37	10.94	16.60

Note: Values are presented as mean ± standard deviation. Abbreviations: MAS, modified Ashworth scale; SEM, standard error measurement; MDC, minimal detectable change; 5xSTS, 5-times sit to stand test; F8WT, figure of 8 walk test; FSST, four square step test.

## Data Availability

The data presented in this study are available on request from the corresponding author.

## Abbreviations

ABC scale	Activities-specific Balance Confidence Scale
5xSTS	Five Times Sit-to-Stand Test
F8WT	Figure-of-8 Walk Test
FSST	Four-Square Step Test
MDC	Minimal detectable change
TUG	Timed Up and Go Test
ICC	Intraclass correlation coefficient
MAS	Modified Ashworth Scale
SEM	Standard error of measurement

## References

[1] Tang A., Rymer W.Z. (1981). Abnormal Force--EMG Relations in Paretic Limbs of Hemiparetic Human Subjects. J. Neurol. Neurosurg. Psychiatry.

[2] Bourbonnais D., Vanden Noven S. (1989). Weakness in Patients with Hemiparesis. Am. J. Occup. Ther..

[3] Sommerfeld D.K., Eek E.U.-B., Svensson A.-K., Holmqvist L.W., von Arbin M.H. (2004). Spasticity After Stroke. Stroke.

[4] Urban P.P., Wolf T., Uebele M., Marx J.J., Vogt T., Stoeter P., Bauermann T., Weibrich C., Vucurevic G.D., Schneider A. (2010). Occurence and Clinical Predictors of Spasticity after Ischemic Stroke. Stroke.

[5] Shumway-Cook A., Woollacott M.H. (2001). Motor Control: Theory and Practical Applications.

[6] Watkins C.L., Leathley M.J., Gregson J.M., Moore A.P., Smith T.L., Sharma A.K. (2002). Prevalence of Spasticity Post Stroke. Clin. Rehabil..

[7] Bressel E., McNair P.J. (2002). The Effect of Prolonged Static and Cyclic Stretching on Ankle Joint Stiffness, Torque Relaxation, and Gait in People with Stroke. Phys. Ther..

[8] Selles R.W., Li X., Lin F., Chung S.G., Roth E.J., Zhang L.-Q. (2005). Feedback-Controlled and Programmed Stretching of the Ankle Plantarflexors and Dorsiflexors in Stroke: Effects of a 4-Week Intervention Program. Arch. Phys. Med. Rehabil..

[9] Taylor M.J.D., Strike S.C., Dabnichki P. (2006). Strategies Used for Unconstrained Direction Change during Walking. Percept. Mot. Skills.

[10] Glaister B.C., Bernatz G.C., Klute G.K., Orendurff M.S. (2007). Video Task Analysis of Turning during Activities of Daily Living. Gait Posture.

[11] Powell L.E., Myers A.M. (1995). The Activities-Specific Balance Confidence (ABC) Scale. J. Gerontol. A Biol. Sci. Med. Sci..

[12] Whitney S.L., Wrisley D.M., Marchetti G.F., Gee M.A., Redfern M.S., Furman J.M. (2005). Clinical Measurement of Sit-to-Stand Performance in People with Balance Disorders: Validity of Data for the Five-Times-Sit-to-Stand Test. Phys. Ther..

[13] Hess R.J., Brach J.S., Piva S.R., VanSwearingen J.M. (2010). Walking Skill Can Be Assessed in Older Adults: Validity of the Figure-of-8 Walk Test. Phys. Ther..

[14] Dite W., Temple V.A. (2002). A Clinical Test of Stepping and Change of Direction to Identify Multiple Falling Older Adults. Arch. Phys. Med. Rehabil..

[15] Salbach N.M., Mayo N.E., Higgins J., Ahmed S., Finch L.E., Richards C.L. (2001). Responsiveness and Predictability of Gait Speed and Other Disability Measures in Acute Stroke. Arch. Phys. Med. Rehabil..

[16] Cheng D.K.-Y., Dagenais M., Alsbury-Nealy K., Legasto J.M., Scodras S., Aravind G., Takhar P., Nekolaichuk E., Salbach N.M. (2021). Distance-Limited Walk Tests Post-Stroke: A Systematic Review of Measurement Properties. NeuroRehabilitation.

[17] Podsiadlo D., Richardson S. (1991). The Timed “Up & Go”: A Test of Basic Functional Mobility for Frail Elderly Persons. J. Am. Geriatr. Soc..

[18] Schuck P., Zwingmann C. (2003). The “smallest Real Difference” as a Measure of Sensitivity to Change: A Critical Analysis. Int. J. Rehabil. Res..

[19] Chen H.-M., Hsieh C.-L., Liaw L.-J., Chen S.-M., Lin J.-H. (2007). The Test-Retest Reliability of 2 Mobility Performance Tests in Patients with Chronic Stroke. Neurorehabilit. Neural Repair.

[20] Steffen T., Seney M. (2008). Test-Retest Reliability and Minimal Detectable Change on Balance and Ambulation Tests, the 36-Item Short-Form Health Survey, and the Unified Parkinson Disease Rating Scale in People with Parkinsonism. Phys. Ther..

[21] Beckerman H., Roebroeck M.E., Lankhorst G.J., Becher J.G., Bezemer P.D., Verbeek A.L. (2001). Smallest Real Difference, a Link between Reproducibility and Responsiveness. Qual. Life Res..

[22] Smidt N., van der Windt D.A., Assendelft W.J., Mourits A.J., Devillé W.L., de Winter A.F., Bouter L.M. (2002). Interobserver Reproducibility of the Assessment of Severity of Complaints, Grip Strength, and Pressure Pain Threshold in Patients with Lateral Epicondylitis. Arch. Phys. Med. Rehabil..

[23] Hiengkaew V., Jitaree K., Chaiyawat P. (2012). Minimal Detectable Changes of the Berg Balance Scale, Fugl-Meyer Assessment Scale, Timed “Up & Go” Test, Gait Speeds, and 2-Minute Walk Test in Individuals with Chronic Stroke with Different Degrees of Ankle Plantarflexor Tone. Arch. Phys. Med. Rehabil..

[24] Mahmoudzadeh A., Nakhostin Ansari N., Naghdi S., Ghasemi E., Motamedzadeh O., Shaw B.S., Shaw I. (2021). Role of Spasticity Severity in the Balance of Post-Stroke Patients. Front. Hum. Neurosci..

[25] Radinmehr H., Ansari N.N., Naghdi S., Tabatabaei A., Moghimi E. (2019). Comparison of Therapeutic Ultrasound and Radial Shock Wave Therapy in the Treatment of Plantar Flexor Spasticity After Stroke: A Prospective, Single-Blind, Randomized Clinical Trial. J. Stroke Cerebrovasc. Dis..

[26] Botner E.M., Miller W.C., Eng J.J. (2005). Measurement Properties of the Activities-Specific Balance Confidence Scale among Individuals with Stroke. Disabil. Rehabil..

[27] Ishige S., Wakui S., Miyazawa Y., Naito H. (2020). Reliability and Validity of the Activities-Specific Balance Confidence Scale-Japanese (ABC-J) in Community-Dwelling Stroke Survivors. Phys. Ther. Res..

[28] Muñoz-Bermejo L., Adsuar J.C., Mendoza-Muñoz M., Barrios-Fernández S., Garcia-Gordillo M.A., Pérez-Gómez J., Carlos-Vivas J. (2021). Test-Retest Reliability of Five Times Sit to Stand Test (FTSST) in Adults: A Systematic Review and Meta-Analysis. Biology.

[29] Leigh Hollands K., Hollands M.A., Zietz D., Miles Wing A., Wright C., van Vliet P. (2010). Kinematics of Turning 180° During the Timed Up and Go in Stroke Survivors With and Without Falls History. Neurorehabilit. Neural Repair.

[30] Kuan Y.-C., Lin L.-F., Wang C.-Y., Hu C.-C., Liang P.-J., Lee S.-C. (2022). Association Between Turning Mobility and Cognitive Function in Chronic Poststroke. Front. Neurol..

[31] Blennerhassett J.M., Jayalath V.M. (2008). The Four Square Step Test Is a Feasible and Valid Clinical Test of Dynamic Standing Balance for Use in Ambulant People Poststroke. Arch. Phys. Med. Rehabil..

[32] Hong S.-J., Goh E.Y., Chua S.Y., Ng S.S. (2012). Reliability and Validity of Step Test Scores in Subjects with Chronic Stroke. Arch. Phys. Med. Rehabil..

[33] Lord S.E., McPherson K., McNaughton H.K., Rochester L., Weatherall M. (2004). Community Ambulation after Stroke: How Important and Obtainable Is It and What Measures Appear. Predictive?. Arch. Phys. Med. Rehabil..

[34] Levin M.F., Hui-Chan C.W. (1992). Relief of Hemiparetic Spasticity by TENS Is Associated with Improvement in Reflex and Voluntary Motor Functions. Electroencephalogr. Clin. Neurophysiol..

[35] Kim S.-M., Kang S.-H. (2021). The Effects of Task-Oriented Circuit Training Using Unstable Surface on Balance, Walking and Balance Confidence in Subacute Stroke Patients. J. Korean Soc. Integr. Med..

[36] Lakens D. (2013). Calculating and Reporting Effect Sizes to Facilitate Cumulative Science: A Practical Primer for t-Tests and ANOVAs. Front. Psychol..

[37] Gregson J.M., Leathley M.J., Moore A.P., Smith T.L., Sharma A.K., Watkins C.L. (2000). Reliability of Measurements of Muscle Tone and Muscle Power in Stroke Patients. Age Ageing.

[38] Mong Y., Teo T.W., Ng S.S. (2010). 5-Repetition Sit-to-Stand Test in Subjects with Chronic Stroke: Reliability and Validity. Arch. Phys. Med. Rehabil..

[39] Wong S.S.T., Yam M.-S., Ng S.S.M. (2013). The Figure-of-Eight Walk Test: Reliability and Associations with Stroke-Specific Impairments. Disabil. Rehabil..

[40] Goh E.Y., Chua S.Y., Hong S.-J., Ng S.S. (2013). Reliability and Concurrent Validity of Four Square Step Test Scores in Subjects with Chronic Stroke: A Pilot Study. Arch. Phys. Med. Rehabil..

[41] Portney L.G., Watkins M.P. (2009). Foundations of Clinical Research: Applications to Practice.

[42] Bourke A.K., Scotland A., Lipsmeier F., Gossens C., Lindemann M. (2020). Gait Characteristics Harvested during a Smartphone-Based Self-Administered 2-Minute Walk Test in People with Multiple Sclerosis: Test-Retest Reliability and Minimum Detectable Change. Sensors.

[43] English C., Hillier S.L., Lynch E.A. (2017). Circuit Class Therapy for Improving Mobility after Stroke. Cochrane Database Syst. Rev..

[44] Jin Y., Lee Y., Park S., Lee S., Lim C. (2023). Effects of Curved-Path Gait Training on Gait Ability in Middle-Aged Patients with Stroke: Protocol for a Randomized Controlled Trial. Healthcare.

[45] Heeren A., van Ooijen M., Geurts A.C.H., Day B.L., Janssen T.W.J., Beek P.J., Roerdink M., Weerdesteyn V. (2013). Step by Step: A Proof of Concept Study of C-Mill Gait Adaptability Training in the Chronic Phase after Stroke. J. Rehabil. Med..

[46] Timmermans C., Roerdink M., Meskers C.G.M., Beek P.J., Janssen T.W.J. (2021). Walking-Adaptability Therapy after Stroke: Results of a Randomized Controlled Trial. Trials.

[47] Mehrholz J., Thomas S., Elsner B. (2017). Treadmill Training and Body Weight Support for Walking after Stroke. Cochrane Database Syst. Rev..

[48] Marigold D.S., Eng J.J. (2006). The Relationship of Asymmetric Weight-Bearing with Postural Sway and Visual Reliance in Stroke. Gait Posture.

[49] Sosnoff J.J., Shin S., Motl R.W. (2010). Multiple Sclerosis and Postural Control: The Role of Spasticity. Arch. Phys. Med. Rehabil..

[50] Rahimzadeh Khiabani R., Mochizuki G., Ismail F., Boulias C., Phadke C.P., Gage W.H. (2017). Impact of Spasticity on Balance Control during Quiet Standing in Persons after Stroke. Stroke Res. Treat..

[51] Weiss A., Suzuki T., Bean J., Fielding R.A. (2000). High Intensity Strength Training Improves Strength and Functional Performance after Stroke. Am. J. Phys. Med. Rehabil..

[52] Lomaglio M.J., Eng J.J. (2005). Muscle Strength and Weight-Bearing Symmetry Relate to Sit-to-Stand Performance in Individuals with Stroke. Gait Posture.

[53] Ng S.S., Hui-Chan C.W. (2005). The Timed up & Go Test: Its Reliability and Association with Lower-Limb Impairments and Locomotor Capacities in People with Chronic Stroke. Arch. Phys. Med. Rehabil..

[54] Ng S.S.M., Shepherd R.B. (2000). Weakness in Patients with Stroke: Implications for Strength Training in Neurorehabilitation. Phys. Ther. Rev..

[55] Ng S.S.M., Ng P.C.M., Lee C.Y.W., Ng E.S.W., Tong M.H.W. (2012). Walkway Lengths for Measuring Walking Speed in Stroke Rehabilitation. J. Rehabil. Med..

[56] Ng S.S.M., Hui-Chan C.W.Y. (2013). Ankle Dorsiflexor, Not Plantarflexor Strength, Predicts the Functional Mobility of People with Spastic Hemiplegia. J. Rehabil. Med..

[57] Hsueh I.-P., Hsu M.-J., Sheu C.-F., Lee S., Hsieh C.-L., Lin J.-H. (2008). Psychometric Comparisons of 2 Versions of the Fugl-Meyer Motor Scale and 2 Versions of the Stroke Rehabilitation Assessment of Movement. Neurorehabilit. Neural Repair..

